# Perceptions and Attitudes towards Medication Error Reporting in Primary Care Clinics: A Qualitative Study in Malaysia

**DOI:** 10.1371/journal.pone.0166114

**Published:** 2016-12-01

**Authors:** A. Samsiah, Noordin Othman, Shazia Jamshed, Mohamed Azmi Hassali

**Affiliations:** 1 Kulliyyah of Pharmacy, International Islamic University Malaysia, Kuantan, Pahang, Malaysia; 2 Institute for Health Systems Research, Ministry of Health, Shah Alam, Selangor, Malaysia; 3 Department of Clinical and Hospital Pharmacy, College of Pharmacy, Taibah University, Almadinah Almunawwarah, KSA; 4 School of Pharmaceutical Sciences, University Sains Malaysia, Penang, Malaysia; Universita degli Studi di Padova, ITALY

## Abstract

**Objective:**

To explore and understand participants’ perceptions and attitudes towards the reporting of medication errors (MEs).

**Methods:**

A qualitative study using in-depth interviews of 31 healthcare practitioners from nine publicly funded, primary care clinics in three states in peninsular Malaysia was conducted for this study. The participants included family medicine specialists, doctors, pharmacists, pharmacist assistants, nurses and assistant medical officers. The interviews were audiotaped and transcribed verbatim. Analysis of the data was guided by the framework approach.

**Results:**

Six themes and 28 codes were identified. Despite the availability of a reporting system, most of the participants agreed that MEs were underreported. The nature of the error plays an important role in determining the reporting. The reporting system, organisational factors, provider factors, reporter’s burden and benefit of reporting also were identified.

**Conclusions:**

Healthcare practitioners in primary care clinics understood the importance of reporting MEs to improve patient safety. Their perceptions and attitudes towards reporting of MEs were influenced by many factors which affect the decision-making process of whether or not to report. Although the process is complex, it primarily is determined by the severity of the outcome of the errors. The participants voluntarily report the errors if they are familiar with the reporting system, what error to report, when to report and what form to use.

## Introduction

Errors in healthcare practice are primarily the results of weaknesses in work systems [1]. To formulate appropriate safety solutions, the errors and their causes need to be identified and understood. This can be achieved through reporting of errors through any local or national reporting system as one of the tools to detect current issues of patients’ safety [1]. The goals of error reporting systems are learning and sharing as well as exchanging of information from healthcare past failures to enhance patients’ safety [2]. The key to reaching these goals is the reporting of errors through a national reporting system which enables sharing of lessons learnt to an extensive target audience [3].

Medication errors (ME) are among the most common errors in health care which may cause negative consequences to the patients’ outcome. In response to above recommendation, numerous ME reporting tools have been constructed nationally or locally, in many countries and healthcare organisations. Although some countries have adopted a voluntary system, others practise a mandatory type of system; some systems are designed for MEs, while others are for multiple errors [4–11]. The Malaysia Ministry of Health (MOH) introduced the Medication Error Reporting System (MERS) in August 2009. MERS is a national reporting system for ME with the objective of collecting information and maintaining a proper database. The database will be analysed and used as the foundation for proposing appropriate remedial actions [5].

Despite the positive development in reporting systems, underreporting of MEs persists and remains prevalent. Previous studies have documented that healthcare practitioners (HCPs) believed the percentage of MEs reported was far less than the actual occurrences. The estimated percentage of MEs reported for nurses in different health care settings was 28.9% [12], less than 60% [13], 40% or less [14] and around 50% [15], [16]. A recent study among nurses in a tertiary hospital in Saudi Arabia suggested that up to 79% of actual errors were not reported [17].

The underreporting of MEs has been associated with barriers to reporting. Among the reasons cited were being unsure of what and how to report [18–21], increased burden of effort for the HCPs [18], [19], [22], [23], organisational factors such as lack of feedback [21], [24], [25], blaming the individual instead of the system [14], [16], [17] and fear-related factors [13], [16], [26], [27].

Although a majority of studies mostly concentrated on the barriers to reporting, some studies highlighted factors facilitated error reporting. The simplicity of the reporting form [18], [22], adequate training on the reporting process [18], anonymous reporting [22], [28], adequate feedback received on the reporting [18], [22], perceived severity of error [18], [22], [28] and supportive and open working environment [29], [30] were among the reasons deemed to increase the likelihood of reporting MEs.

A study of community pharmacies using hypothetical MEs indicated that generally the pharmacists and support staffs were unlikely to report errors that occurred at their settings [31]. They deliberate on the error consequences to the patient and the type of behaviour associated with the error while they contemplate whether or not to make an internal or external report about the mistake.

Another study of doctors, nurses and pharmacists of a hospital showed that nurses and pharmacists were more likely to report all types of ME while the doctors were more inclined to report serious errors compared with near misses [32].

Previous studies examining ME reporting mainly focused on hospitals and specialty settings. Less is known about reporting in primary care facilities even though the probability of MEs is high in this setting. A recent literature review on medication safety in Australia reported that 8.5–12% of people who attended the general practice experienced adverse medication effects in the past six months [33].

Similarly, the incidence of MEs reported was high in Malaysian public primary care clinics. Analysis of medical records in randomly selected clinics in 2007 revealed that MEs were detected in 41.1% of the management errors. Two most common types of ME identified were wrong dosage or frequency of medication given (48.5%) and inappropriate medication prescribed (47.2%) [34]. Another locally published study on the perception of doctors and pharmacists towards ME reporting and prevention in primary outpatient clinics showed that both professions perceived that individual blaming is a barrier to reporting but not the heavy workload [35].

Analysis of MEs reported to MERS from 2009 to 2012 discovered that only 16% of the total errors (*N* = 17,357) were reported by the government clinics [36]. The low reporting rate warrants further investigation of why reporting is not occurring among the HCPs in primary care settings.

### Objective of the Study

This study aims to explore and understand healthcare practitioners’ perceptions towards underreporting of MEs, what facilitates and what hinders the reporting of MEs, as well as the attitude of HCPs in reporting self- and peer-caused errors.

## Materials and Methods

### Data Collection

#### Study Design

We deemed in-depth qualitative interviews to be the most suitable method for data collection given the research questions: What are HCPs’ perceptions towards ME reporting, what are the barriers to and facilitators for reporting, and what are HCP’s attitudes in reporting self-caused and peer-caused errors.

#### Recruitment of Participants

Thirty-six HCPs from nine government-funded, primary care clinics in four districts were recruited for the study. Convenience sampling was used to identify potential participants. An official letter enclosed with a suggested schedule of interviews was sent to health district officers to request potential participants from different backgrounds to participate in the interviews. Upon agreement, the health district officers requested the senior officer of each category of HCP to nominate participants and informed the researcher.

The first author carried out the interviews in a closed room at the participants’ respective clinics during office hours in February to April 2014. Of the 36 recruited, five participants were unable to participate. One pharmacist and two family medicine specialists (FMS) were attending courses, while two pharmacist assistants were staffing the counter due to a lack of staff on the day the interviews were scheduled.

#### Interview Sessions

A semi-structured interview guide (S1 Appendix) was developed as the study instrument based on a literature review of similar studies [18], [22]. The interview guide was pre-tested with a FMS and two assistant medical officers.

A total of 21 in-depth, face-to-face interview sessions were conducted either individually or in a small group of participants with similar professions. These methods were chosen because they fit the participants’ working schedule and enabled them to freely express their opinions.

Prior to the interview, participants were briefed about the objectives and expectations of the study. Next, they completed an individual written consent form and demographic data table. The interviews were audiotaped upon their permission. Each interview took 45–60 minutes and was conducted either in English or the Malay language. Each participant was assigned a unique pseudonym for confidentiality purposes [37]. Appropriate probes were used to stimulate more information about a particular issue [38]. At the end of the interview, each participant received a small token of appreciation.

The saturation point was reached after conducting 16 interviews with 25 participants from all backgrounds. At this stage, no new theme emerged, and data collection was concluded [39]. Another five sessions confirmed this conclusion.

#### Ethical Considerations

The Medical Research Ethic Committee Ministry of Health Malaysia (NMRR-13-1219-17743) and International Islamic University of Malaysia Research Ethic Committee (IREC 162) approved this study. Permission also was obtained from the directors of the Health State Department and the respective district health officers. Each participant signed a consent form before the interview.

### Data Analysis

Data analysis was guided by the framework approach [40], [41]. The procedure started with the verbatim transcription of the interviews. Transcriptions in Malay language were translated into English by professional translators. Next was the familiarisation stage in which the audio recording of each interview was listened to whilst the transcription was being read.

Two researchers then independently examined a few transcripts line by line and assigned codes to denote particular meaningful segments. This coding combined both deductive and inductive approaches. Once the coding for the first few transcripts was completed, the two researchers compared the coding and agreed on a set of codes to be applied to all subsequent transcripts. The codes were grouped into categories or themes. These formed a working analytical framework which was based on the data and a prior understanding of the literature. The two researchers discussed any disagreements. The segmented data were coded after several readings for the final coding. A third researcher acted as a referee if the disagreements were not resolved.

The working analytical framework then was applied to the remaining transcripts. New codes were added into the working framework throughout the process of data analysis. The initial framework was reviewed by deleting or merging the codes appropriately. The framework was refined further to improve clarity and reduce ambiguity to produce the final thematic framework.

Subsequently, the data were charted into the framework matrix which involved summarizing data by category for each transcript. The matrix enabled the researchers to make final conclusions regarding the rich data. Upon completion, the framework was updated with six categories or themes and 28 codes.

## Results

### Characteristics of Participants

Table 1 presents the characteristics of the participants (*n* = 31). The sample was comprised of 18 females and 13 males. The number of years of experience ranged from three months to 16 years. The participants consisted of family medicine specialists, doctors, pharmacists, pharmacist assistants, nurses and assistant medical officers. They represented the whole category of frontline HCPs in a position to involve, detect, report and manage MEs.

**Table 1:** Characteristics of Participants.

**Profession**	**Male**	**Female**
Family Medicine Specialist	1	1
Doctor	2	2
Pharmacist	3	5
Pharmacist Assistant	1	4
Nurse	0	6
Assistant Medical Officer	6	0
**Duration of practice experience (in primary care clinic)**		
≤ 1 year	5	
> 1 year to 5 years	8	
≥ 5 years to 10 years	12	
> 10 years	6	

### General Findings

All participants agreed that MEs occurred in their work place. The majority of participants understood the definitions of ME error and ME reporting. They also were aware that MEs had to be reported.

The severity of the ME outcome was the deciding factor for reporting MEs, as this was the main issue discussed in every interview. According to the participants, multiple reporting systems for MEs are readily available at primary care clinics, namely Incident Reporting (IR), Quality Assurance Programme (QAP), Pharmacy Statistics (PF), Daily Activity Logbook, Medication Error Reporting System (MERS) and verbal reporting.

MERS, as the official reporting method for MEs for all healthcare facilities in Malaysia including the private sector, is well known to pharmacists. Unfortunately, nearly all the other categories of participants were unaware of its existence.

The majority of HCPs agreed that MEs were underreported at their respective clinic. The perceived underreporting was relatively severe. The findings also revealed that the practice of reporting MEs using MERS, either manually or online, was very minimal. In some clinics, the percentage of MEs reported was extremely low. Only a few participants reported an ME through any means within the past 6–12 months.

### Thematic Framework

Six main themes emerged from the analysis. Table 2 outlines the themes, their associated codes complete with number and profession of the HCPs’ responded.

**Table 2:** Thematic Framework.

Theme/Category	Codes	No of HCPs responded (N = 31)	Profession
Nature of error	Severity of outcome	29	all categories
	Type of drug	4	doctor, pharmacist, assistant medical officer
	Frequency of errors	8	FMS, doctor, pharmacist, pharmacist assistant
	Unimportant errors	7	doctor, pharmacist, pharmacist assistant, assistant medical officer
	Repetition of errors	8	doctor, pharmacist, pharmacist assistant, assistant medical officer
Reporting system	Reporting mode		
	• MERS (online and/or paper-based)	5	pharmacist
	• PF and/or QAP form	9	pharmacist, pharmacist assistant
	• Incident Reporting	7	FMS, doctor, assistant medical officer, nurse
	• Daily activity log book	4	nurse
	• Verbal	5	doctor, assistant medical officer, pharmacist assistant
	Confidentiality	4	doctor, pharmacist, pharmacist assistant
	Reporting form	5	doctor, pharmacist, nurse, pharmacist assistant
	Targeted reporting	5	pharmacist
Organisational factors	Education and training	21	all categories
	Push factor	9	pharmacist, pharmacist assistant, assistant medical officer, nurse
	Dedicated officer	5	doctor, pharmacist, assistant medical officer
	Feedback	10	FMS, doctor, pharmacist
	Maintain professional relationship	4	pharmacist, assistant medical officer
	Maintain clinic reputation	3	doctor, pharmacist, assistant medical officer
	Reporting culture		
	• Blaming culture	2	pharmacist, assistant medical officer
	• Concern of superiors’ reaction	4	pharmacist, nurse
	• Concern of repercussion from patient	3	FMS, doctor
	• Concern of colleague’s reaction	1	pharmacist assistant
	• Concern others will know	2	nurse, pharmacist assistant
	• Concern of getting low mark in annual performance appraisal	2	pharmacist assistant
	• Concern of being labelled as incompetent staff	1	doctor
Reporter’s burden	Overlapping reporting	6	pharmacist, pharmacist assistant
	Workload pressure	10	FMS, doctor, pharmacist, assistant medical officer
	Shortage of staff	4	doctor, pharmacist, pharmacist assistant
	Huge paperwork	5	pharmacist
Provider related factors	Unknowingness	13	FMS, doctor, pharmacist assistant, assistant medical officer, nurse
	Role in reporting	21	all categories
	Responsibility	16	all categories
	Routine task	4	nurses
Benefit from reporting	Change in practise	18	all categories
	Prevent reoccurrence	8	FMS, doctor, pharmacist, assistant medical officer, nurse
	Self-protection	7	doctor, pharmacist, assistant medical officer, pharmacist assistant, nurse
	Vigilance	5	FMS, assistant medical officer, pharmacist assistant, nurse

#### Nature of Error

Nature of error was the main theme identified from the interviews. It was raised by interviewees of all categories. The majority of the interviewees agreed that the severity of ME outcome was the key factor which influenced the attitude to report an error they committed or a peer committed. They said that theoretically both actual errors and near misses should be reported as this will portray a complete picture of all sources of risks and events harmful to patients. Additionally, an FMS said that ME cases with severe outcomes which have the potential for medical-legal action should be reported for the benefit of both patients and HCPs.

*All error should be reported but in health clinic we have other workload so we chose to report error which caused harm*.*… But for minor cases such as wrong dose prescribed will not report*.

(*Pharmacist, Interview 8*)

*In my opinion*, *both the near misses and actual errors should be reported*, *unless it is just a minor mistake such as illegible handwriting…anything near misses or actual errors should be reported so as to prevent further mistakes and to increase the awareness among the staff*.

(*Doctor, Interview 19*)

MEs that involved chronic drugs such as hypertensive medications were more likely to be reported than drugs for mild conditions such as cough and cold. Errors pertaining to injectable and vaccination drugs were considered very critical for reporting. Such cases would be investigated, the contributing factors identified and appropriate actions executed to alert others so as to prevent reoccurrence. The following quote supported this argument.

*In my experience…it is more on vaccination…wrongly given or double doses*.*… It is a serious matter… it will be reported and investigated*.*… Wrong vaccination or injections will make everyone alert…They will interview everyone to make sure it will not happen again*.*… But for oral medication…we rarely heard about such things… such as wrongly given…but vaccination yes…that is in the primary level*.

(*Doctor, Interview 18*)

The high frequency of near misses contributed to low reporting. Participants believed that near misses were harmless or that the near misses were detected before they reached the patients, hence immediate action could be taken, making reports unnecessary. A few participants, however, suggested that near misses should be monitored at an internal level so that immediate action could be taken and the individual concerned should be advised and re-educated.

*If you say that near misses in prescription such as writing wrongly as what the pharmacy called it…we have to report…probably we will not report all of them because there are many*.*…But for serious event*, *nearly wrongly injected that rarely happened probably we will report*.

(*Doctor, Interview 18*)

On the other hand, a couple of participants believed that primary care clinics handled fewer chronic cases and fewer medications were prescribed, meaning there were fewer chances for MEs to occur and a low reporting of MEs at these settings.

Errors which participants considered as trivial—especially those involving prescriptions errors such as the wrong duration, wrong dosage and incomplete patient data—were unlikely to be reported. These types of errors can be resolved through communication with the prescribers or patients at the point the errors occurred.

*…I know you can actually report the near misses but cases like wrong duration…all those to me are small problems…we can just re-confirm with the patients or the prescribers…This is more like a serious error*, *I would say…or error occurred then I will fill in the form*…

(*Pharmacist, Interview 9*)

*The reason being [for not reporting near misses error] was that the problem can be settled at that time*.

(*Pharmacist Assistant, Interview 14*)

Some participants viewed that repetition of the same errors or by the same individual as facilitators for reporting.

#### Reporting System

Manual mechanism is the main method used to report MEs in primary care clinics. MERS, QAP and PF are used mainly by the pharmacy staff while other HCPs used IR. The Daily Activity Logbook is practised in the nursing department. Verbal method is still used in a few clinics.

MERS was upgraded to an online system recently and among all the clinics represented by the interviewees, only one is using it. The pharmacists from the other five clinics knew about the online MERS system but hadn’t yet used it. The efficiency of the online MERS system depended mostly on accessibility to it.

*Yes…using MERS online…*.*so far this year we reported 3 events using MERS online…with the online system it was quite ok but still depending on time and internet connection*…

(*Pharmacist, Interview 8*)

The participants wish their confidentiality to be protected if they make a report. If their confidentiality is guaranteed, they are motivated to report ME events.

*Or maybe because we do not want to expose the identity of individual who committed the error*…

(*Pharmacist Assistant, Interview 4*)

*If we know our information is kept confidential*, *then we will report*.

(*Doctor, Interview 18*)

Participants said a simplified reporting form would encourage the HCPs to submit more reports. They believed a streamlined form could be completed faster and comprehended better, saving time and being less troublesome. A pharmacist expressed his satisfaction with the online MERS which he considered convenient.

*Before this…it is because of the reporting form is tedious…we have to tick everything…we have to fill the error part…there are so many boxes to tick…we have no time…but now with the online system it was quite alright …*.

(*Pharmacist, Interview 8*)

More than half of the pharmacists recommended a specific target to be fixed to increase the number of MEs reports.

*Unlike other activities…for example counselling*, *we must do at least 2 sessions per day…so far for ME there is no such target…not in our annual target*…

(*Pharmacist, Interview 8*)

#### Organisational Factors

Almost all categories of participants voiced their need for more education and training on all aspects of ME reporting. They felt that training should cover a wide array of HCPs rather than focusing only on the pharmacists. Other HCPs expressed their willingness to report provided they understood the significance of the reporting and were familiar with reporting process.

*It would be good if there is one yearly or more frequent seminars or talks regarding ME…whether it is from the pharmacy side or our superior…it should be in details on how to report and what happen after the formal reporting*, *not only for doctors but for nurses and Assistant Medical Officers as well*.

(*Doctor, Interview 19*)

*To me…we all are less exposed…we don’t quite understand how to fill up the reporting form*, *how to report…If possible arrange a talk…give us enough exposure…so we will know what to do next*…

(*Pharmacist Assistant, Interview 7*)

Any form of push factor (e.g. encouragement, enforcement or emphasis on the importance of reporting MEs) was viewed as another strategy to encourage reporting. Otherwise, HCPs would not consider the matter seriously.

*…Someone needs to emphasise on the MERS…or else we will not take it seriously*. *Even for the near misses and others…we have to report*…

(*Pharmacist, Interview 11*)

*Perhaps there is no enforcement…unlike other cases…they have Acts*, *Regulation etc*.

(*Assistant Medical Officer, Interview 2*)

*If you report more…they said it is a good practice…so…just report*.

(*Nurse, Interview 20*)

A few participants pointed out on the importance of having a dedicated officer to coordinate the reporting of MEs. One interviewee, a pharmacist, is the coordinator for reporting MEs at his clinic, which is fairly active in reporting to MERS. This showed that a dedicated officer can contribute a significant role to strengthening the reporting practice.

*I am responsible for reporting any errors…like what I said just now…cases that I reported were major cases…minor cases will not be reported…Yes…for Adverse Effects Following Immunisation (AEFI)*, *ME and Adverse Drug Reactions (ADR)*, *I am responsible to report…actually anybody can report…but I am the one who compiled all the reports*.

(*Pharmacist, Interview 8*)

*At my previous workplace*, *the highest grade pharmacist in the district is assigned as the coordinator…he will coordinate all reports…and the reports will be sent to Health District Office and State Health Department*.

(*Assistant Medical Officer, Interview 16*)

Study findings showed that participants learnt about ME and ME reporting either through informal or formal training. Some of them read about MEs from printed materials. Many of the interviewees gained knowledge through work experience and informal discussions with peers.

Nevertheless, most of the FMSs, doctors and pharmacists disclosed that they lacked feedback from the reported ME. They expressed their curiosity on their respective clinic performance as well as the whole scenario of MEs reported nationwide.

*…because we don’t know…when we report what did MOH do with it…I have not seen any report or feedback from the MOH…we did not see any…we never know how many reports are there from our state…in a year how many reports and feedback from each state…so that we can see our own performance…But I don’t know…If others report then it will encourage us to report too*.

(*Pharmacist, Interview 12*)

*To my knowledge*, *we as the prescriber are more to prescribing*. *There are cases out there…the pharmacist or assistant wrongly dispense the medication…even though the prescription is correct*. *That is beyond my knowledge…Even if it did happen*, *I wouldn’t know because it wasn’t discussed*, *so far there is no avenue for discussion*. *Maybe the pharmacists discussed the incident among themselves but not with us*…

(*FMS, Interview 1*)

A closed relationship among the clinic staff increased the likelihood of reporting MEs. They try to maintain a harmonious professional relationship with colleagues with dissimilar backgrounds. A pharmacist mentioned that subsequent to reporting, an urgent meeting would be called to discuss the reported error. This created uncomfortable situation among the HCPs.

*We work here together every day, so we want to have a smooth relationship with the other staffs… If we make reports to the higher level, there will be special meetings and then, they will ask ‘why did the pharmacy want to report such small cases?*’

(*Pharmacist, Interview 3*)

*Not because we do not want to report…but we want to maintain a good internal relationship between the pharmacy and related officers*.

(*Assistant Medical Officer, Interview 17*)

A few participants raised the issue of upholding the clinic reputation as well as the quality services as one of the reasons for not reporting.

*They want to maintain the clinic’s standard*.

(*Assistant Medical Officer, Interview 2*)

A few participants also commented on the culture of reporting MEs in primary care clinics. They expressed concern over the reaction of others including their superiors, colleagues and patients or their families. They believed they would be blamed and punished by their bosses. They felt embarrassed when others knew they committed errors as they would be labelled as incompetent and will ruin their reputation. As a consequence, they feared that these reports somehow would affect their annual performance appraisal.

These concerns were considered minor factors, however, and did not stop them from reporting because patients’ safety remained a top priority. They felt more concern if a harmful error was not reported rather than fear of any action imposed upon them.

*Reporting will be an issue if the reporter feel that they do not have any advantage…no benefit…they feel they are blamed…If we want to strengthened MERS*, *reporters should be protected…there must be a guarantee…when someone make a report*, *he or she should not be blamed*.

(*Assistant Medical Officer, Interview 16*)

*I think it is mostly on the disciplinary action*, *because if the ME is life threatening that is quite a serious case*. *But my personal view is*, *if it involves patients*, *any mistake*, *minor or major should not happened…My personal fear is the disciplinary action…but it is just a minor factor*, *not a major factor*.

(*Medical Officer, Interview 19*)

*Fear to report…Maybe for some people this is true…they do not want others to know and to talk about the incident…The patients will be affected*.

(*Pharmacist Assistant, Interview 4*)

An FMS commented that HCPs should not feel fear to report ME because they will not be blamed and their annual performance will not be affected because of MEs.

*…I don’t think it will be a reason for not reporting…if a staff is fearful because of her mistake…If it is not a big issue…she will not be scolded…If they are wrong then we have to advise them…But they should not worry about getting low marks…because we consider the overall performance not merely based on an error*.

(*FMS, Interview 13*)

#### Reporter’s Burden

Overlapping of reporting MEs to different reporting systems was quoted in many interviews, mainly by the pharmacy staff who is the prime reporters. This overlap stopped them from making a report. A few pharmacists highlighted that they have to report MEs through more than one means at a time: MERS, IR, QAP and PF statistics.

*So many reporting at one time and redundant works… If we fill up MERS we have to fill up IR as well…need to fill up both*…

(*Pharmacist, Interview 9*)

The interviewees stressed that most of their time was focused on patient care. Burdened with the pressure of a heavy workload and shortage of staff, the time for reporting MEs was limited and thus it was given less priority.

*We have many tasks including counselling… and we have to offer VAS [value added service]*, *which we have to brief patients about the service…There are many things to be done at the counter coupled with other workload…ME did happen but not so bad…we assumed that as small mistake*.

(*Pharmacist, Interview 12*)

*One more thing in this clinic is the time factor; the patient load is high and each day we have around 5 to 7 doctors working with all the appointment of chronic illnesses*. *At one time only 2 to 3 doctors available to see the patients*, *that will limit our reporting too*, *because what I know about IR is we need to fill in a lot of things in details …so that will limit the reporting as well*.

(*Doctor, Interview 19*)

According to the interviewees, many of the pharmacists at primary care clinics are new. Also, there are only a few senior staff members and experienced officers at the pharmacy counter in any given district. This caused a lack of guidance for and monitoring of junior staff with regards to the reporting of MEs.

*In the clinic*, *the officers are rather new…they came in and out*. *Unless for clinics with senior officers*, *they can give awareness and guide other staffs on ME…The newly transferred are fresh graduate* …

(*Pharmacist, Interview 5*)

A number of pharmacists mentioned further investigations that were required such as root cause analysis and shortfall in quality procedure once MEs were reported. That such extra forms needed to be completed made them think twice about reporting MEs.

*…when we report ME…there are so many questions…they will ask for so many feedback…there are so many additional task if we make a report*…

(*Pharmacist, Interview 10*)

#### Provider-Related Factors

Adequate knowledge of the reporting system, what to report and when to report eventually lead to successful reporting. When asked to describe the ME reporting system at their work place, only the pharmacists answered correctly. The other categories of participants did not know, were unsure or were confused about the reporting forms.

I learned about the system at the end of last year around October…I don’t know when the system started. …This year…we are using online system… before this we used manual form

(*Pharmacist, Interview 8*)

*I know about reporting Adverse Drug Reaction*, *but for error…I didn’t know that such system for error reporting exists and that we have to report*.

(*FMS, Interview 1*)

According to the interviewees, a lack of knowledge of the existing reporting system and the proper procedure also were barriers to reporting MEs. The unavailability of an ME reporting flowchart made reporting even more difficult.

*There is no flowchart to refer to*.*…If there is one perhaps someone will report…sometimes we want to report but do not know the process…which cases need to be reported are not clear…and what are the categories*.

(*Assistant Medical Officer, Interview 2*)

*We know the error but the reporting flow is not so clear*.

(*Nurse, Interview 6*)

Some of the pharmacist assistants admitted that they were more familiar with QAP and PF forms than the MERS system for reporting MEs.

*Perhaps there is [a system to report ME] but we were not exposed to it…we only know about the intervention form (PF) that we use to jot down the errors*.

(*Pharmacist Assistant, Interview 14*)

All categories of the respondents felt that the duty for reporting MEs should be a responsibility shared among all HCPs. The pharmacist is responsible for completing the form at the pharmacy. Nurses and assistant medical officers inform their supervisor, head of unit or doctor, verbally followed by documenting the incident in the IR book. The higher authority then decides whether or not to proceed with the reporting. However, some institutions have established a committee at district level to manage the reported ME.

A sense of responsibility was found to be one of the facilitators for reporting MEs, a fact which was apparent across all groups of participants. They felt obligated to report an error that most probably has serious implications for the patient’s well-being. This feeling overrode the fear of receiving demerits for having committed the error.

*For example*, *…you wanted to give Measles*, *Mumps and Rubella vaccine but you gave other injection… this one must be reported…the sense of responsibility is bigger than the fear…because we knew that we were careless…we have to be responsible …so we report to our supervisor*.

(*Nurse, Interview 6*)

*One reason is because they feel responsible to report…or else it may cause serious implication to the patient*, *even death…If it is serious…wrong medication may cause death*…

(*FMS, Interview 13*)

Four nurses in two clinics noted that they have been practising the reporting of all patient-related incidents, and this practise has become habitual.

#### Benefit of Reporting

We found that the learning points from reporting were translated to changes in practise at the workplace at both the operational and individual level. Among the highlights were improvements in drug storage and the work station arrangement at the pharmacy. The interviewees also noted the implementation of the 5S system and Tall Man lettering for look-alike and sound-alike medications.

Changes in the medication use process encompassed the whole process, from the prescription of medication to its administration. Interestingly, a pharmacist shared his practise of writing a “pharmacist note” in the patient’s medical record. Any changes to the patient’s drug regimen were written on the record to alert the prescriber and prevent recurrence of MEs. An initiative by the prescribers to reconfirm with patients at the point of prescribing also was practised.

Double checking of medication prepared by two different pharmacy staff members was made compulsory before dispensing. Good dispensing practice was translated by being compassionate towards the patient by asking more questions during the dispensing process if any doubt arose. Use of the 5 Rights principle helped to ensure the right medications were dispensed to the right patient.

*… What I do is that when I dispense medications with other staffs surrounding me… I practise the good dispensing method*, *which helps us to avoid errors…for instance; we have to show that we care about our patients*. *So*, *we will ask the patients more questions…it is not just about the drug*.

(*Pharmacist, Interview 3*).

Although primary care clinics dealt with a minimum of injectable drugs, the individual responsible for administering the medication is to ensure that self-checking was done before the drug was injected. This was mentioned by the assistant medical officers who were directly involved in the drug administration process.

A continuous training session to update the current issue of MEs also was seen as part of a constructive change. This was done through daily reminders and a short training slot to improve and refresh staff members’ awareness and knowledge of MEs.

*I think training helped a lot*. *After the presentation on ME*, *everybody called the pharmacy…to confirm whether the dose was correct…After that we made less mistakes…*.*They reminded us not to write in short form and to write clearly*.

(*Doctor, Interview 18*)

Another notable improvement was a unique job rotation schedule at a pharmacy counter. A two-hour rotation schedule among the pharmacy counter staff was implemented to prevent fatigue due to prolonged and monotonous activity.

We have a schedule here whereby a staff cannot stay at a particular counter for more than 2 hours….which means the staff who dispense, runner and filling will be rotated regularly…It is like a shift work… this is to avoid fatigue…if we keep doing the same task for a long period…once we get tired there is a high possibility to make error.

(Pharmacist Assistant, Interview 7)

Some participants pointed out another reason for reporting an ME was to prevent its recurrence. The participants did not want the same error to recur, and they were optimistic that reporting somehow would solve the problem.

*We reported because we hope that the same mistake won’t happen again*. *We have a responsibility towards the patient; to show that when we make a mistake it doesn’t mean that we don’t care*…

(*Assistant Medical Officer, Interview 2*)

A number of participants anticipated that the reporting of MEs would protect them from any unexpected episodes in the future. They felt safer having complete documentation in cases of serious error.

*No point to hide it…eventually others will know …so it is better to report it immediately…to protect ourselves in the future*. *If the patient wants to sue us or whatever…the event has been reported*.

(*Nurse, Interview 16*)

Meanwhile, some participants reported MEs intending to keep other HCPs in an alert mode about the risk of ME occurrence at their clinics.

## Discussion

This study provides a wide perspective on reporting MEs as viewed by multiple categories of participants involved in different stages of the medication use cycle in primary care clinics: family medicine specialists, doctors, pharmacists, pharmacist assistants, nurses and assistant medical officers. The study examined qualitatively the participants’ perceived barriers and facilitating factors in the reporting of MEs and participants’ attitudes towards reporting errors made by themselves or colleagues and co-workers. Many countries have ME reporting systems at a national or local level [8–11], but to the best of our knowledge studies on the aforementioned objectives in primary care clinics, particularly in Asia, are still lacking.

Most of the participants knew about and understood the importance of reporting MEs but they also were aware that not all MEs were reported due to many factors. According to all categories of participants, the severity of the error outcome for the patient was the key driver to reporting MEs. Harmful or potentially harmful errors were more likely to be reported than MEs perceived to be harmless. This is consistent with other clinically based studies [22], [32], [42], [43]. MEs perceived as unimportant are not reported per a few other studies [14], [24], [44]. Elements such as the type of drugs involved, repetition of errors and frequency of errors also were factors influencing the reporting of MEs [22], as concurred by our findings.

Multiple reporting methods are available at the clinics; however, some of the participants were unclear as to the reporting process, which impeded the reporting activity among interviewed HCPs except for the pharmacists. This finding was parallel with previous studies [12], [21], [25], [45–48]. Participants said a clear flowchart should be made visible to guide the reporting process.

Although many of the participants in this study believed that reporting MEs should be a shared responsibility, the duty rested more on the pharmacists because the majority of the MEs were prescription errors detected at the pharmacy. In interviews conducted with doctors in primary care practices in the United States, the respondents viewed reporting to be the duty of the pharmacists, especially in community health centres with full-time pharmacists [23]. Other HCPs perceived the pharmacists to be a safety net to screen all prescriptions and to contact the prescribers for any discrepancy they detected. When probed further, prescribers said they were unaware that errors had occurred, and a few studies supported these findings [25], [47], [49]. Nevertheless, other HCPs involved in the medication use process should take the initiative to report errors when they receive feedback from the patients regarding the wrongly dispensed medicines.

Based on the interviews, MERS was extremely underutilised in primary care clinics. Almost all of the pharmacists in this study were aware of MERS as the national reporting mechanism for MEs, but it was not the main mode used. Instead, they were more familiar with the QAP or PF mechanisms which have been used for years. Furthermore, they perceived reporting using MERS as a duplicate task. Redundant reporting also was a barrier highlighted by other studies in primary care settings [23], [50]. One way to solve this is by incorporating the reporting activity into the clinician’s current workflow [51].

More education and training activities on ME reporting need to be organised regularly to encourage continuous reporting by all categories of HCPs, which is in line with previous studies [18], [23]. Training on the importance of identifying and reporting MEs can be implemented through many ways including disseminating promotional and educational materials and conducting training sessions for the target population [1].

Participants said they would be more motivated to report MEs if they received feedback on what have been reported. This finding was supported by other studies [22], [51], [52] as it will sustain people’s interests in reporting. Lack of feedback on reported MEs discouraged further reporting [21], [24], [25]. Adequate and timely feedback shows effective communication between administration and staff in sharing errors as a source of education [18], [29]. Feedback not only helps staff to assess their own performance but serves as a platform for sharing and exchanging information on MEs, from within and across organisations [53]. An effective safety feedback system must consider meaningful and timely information to stimulate continuous quality improvements which increase awareness and maintain reporting [54].

A positive culture to reporting and a non-punitive system will encourage reporting [51], [52] while blaming individuals instead of the system will be a barrier [14], [16], [35], [55]. The participants in this study pointed out this issue in hopes that management will focus more on what went wrong in the system rather than who caused the problem. Contrary to previous studies [14], [16], [23], [26], [44], [55], this research found that fear is considered a less important barrier to reporting MEs. Few participants were concerned about disciplinary actions, adverse response from bosses or colleagues, or a negative reaction from the patients and their relatives; this did not deter them from reporting the errors as patient safety is their top priority.

HCPs at the primary care clinics tried to maintain a good professional relationship with each other and were reluctant to report MEs committed by co-workers as it might cause uneasiness and tension among the staff involved. Those who were being reported might feel that the reporters were against them, and the situation could cause their personal working relationship to deteriorate. Williams et al. reported similar findings [22].

A number of notable changes in ME reporting as demonstrated in the study and according to Pham et al. can be used as a measure of a successful reporting system [53]. Physical changes in the arrangement of drug storage and improvement in work processes indicated that the organisation involved learnt from the errors and this consequently led to quality improvement in the work systems.

The study also has several implications over the policy, practice and future research related to this topic. To strengthen and optimise the reporting of MEs, a single reporting mechanism should be enforced. This would directly solve the issue of duplication in reporting MEs. A single database would provide a more accurate data analysis, easy monitoring and a more comprehensive sharing and learning activities.

The types of events that need to be reported and investigated also should be clearly defined. The findings from this study also suggested that more training on reporting of MEs should be conducted among all levels of HCPs in primary care clinics. The situation also requires a more timely and interactive feedback system which serves the real purpose of reporting.

A larger quantitative survey among HCPs in primary care facilities is needed to quantify the perceptions and attitudes towards the reporting of MEs. Finally, further innovative research to enhance the reporting of MEs should be conducted continuously; this in turn could increase the culture of patient safety.

## Limitations

The participants of this study were from a few selected government primary care clinics, thus their views are not representative of HCPs from other health care settings. The number of participants who represented each category of HCPs in this study was small, especially for FMS. We were satisfied, however, because almost all participants had a similar understanding on the definition of MEs according to answers given at the beginning of the interviews. After vigilant investigation, we are certain that no further themes could be retrieved from the generated data, which indicated that the saturation point had been reached.

Due to the staff shortage issue, data collection involved small group and individual face-to-face interviews. A conscientious effort was exercised during the interview to minimise the effect on the findings with appropriate probes or examples in cases that required further explanation or clarification to ensure participants expressed their opinion thoroughly.

## Conclusion

In conclusion, HCPs in primary care health clinics understood the importance of reporting MEs to improve patients’ safety. The decision making for reporting MEs is a complex process but primarily is determined by the severity of the outcome of the errors. Participants voluntarily reported MEs if they understood the reporting system, what error was to be reported, when to report an error and what form should be used.

## Supporting Information

S1 Appendix Semi-structured interview guide. (DOCX)
