# Supplementary material for: Perceptions and Attitudes towards Medication Error Reporting in Primary Care Clinics: A Qualitative Study in Malaysia
Source: PLoS One. 2016 Dec 1;11(12):e0166114. doi: 10.1371/journal.pone.0166114 (PMC5132213; doi:10.1371/journal.pone.0166114)
Supplement: S1 Appendix — (DOCX) [file pone.0166114.s001.docx]

**S1 Appendix. Semi-structured interview guide**

I started each interview by asking the participants to briefly introduce themselves and their role in their respective clinics. The following questions were used to guide the interview. Appropriate prompts were applied to gain further in-depth information when necessary.

1. What do you understand about medication error and medication error reporting?
2. Does your workplace have any reporting system for medication error? Can you describe it?
3. Can you describe what types of medication errors that need to be reported?
4. Who is responsible to report medication errors at your workplace?
5. How do you report a medication error? Are you clear on the reporting process?
6. What is your opinion on the frequency of medication errors reported? What is the estimated reporting percentage/rate of medication errors?
7. Can you share what makes you report the errors? What would encourage you to report more often?
8. In your opinion, why were some of the medication errors not reported?
9. If you make any medication error, what would you do? What makes you decide to report or not to report?
10. If you witness any medication error, what would you do? What make you decide to report or not to report?
11. How often do you report medication errors?
12. In your view, is there any change made based on the error reported?
